# Three-dimensional histopathological reconstruction of bladder tumours

**DOI:** 10.1186/s13000-019-0803-7

**Published:** 2019-03-28

**Authors:** Ilaria Jansen, Marit Lucas, C. Dilara Savci-Heijink, Sybren L. Meijer, Esmee I. M. L. Liem, Onno J. de Boer, Ton G. van Leeuwen, Henk A. Marquering, Daniel M. de Bruin

**Affiliations:** 1 Department of Biomedical Engineering and Physics, Amsterdam UMC, University of Amsterdam, Amsterdam, The Netherlands; 2 Department of Urology, Amsterdam UMC, University of Amsterdam, Amsterdam, The Netherlands; 3 Department of Pathology, Amsterdam UMC, University of Amsterdam, Amsterdam, The Netherlands; 4 Department of Radiology & Nuclear Medicine, Amsterdam UMC, University of Amsterdam, Amsterdam, The Netherlands

**Keywords:** 3D, Bladder cancer; digital pathology, En-bloc, Tumour reconstruction

## Abstract

**Background:**

Histopathological analysis is the cornerstone in bladder cancer (BCa) diagnosis. These analysis suffer from a moderate observer agreement in the staging of bladder cancer. Three-dimensional reconstructions have the potential to support the pathologists in visualizing spatial arrangements of structures, which may improve the interpretation of specimen. The aim of this study is to present three-dimensional (3D) reconstructions of histology images.

**Methods:**

En-bloc specimens of transurethral bladder tumour resections were formalin fixed and paraffin embedded. Specimens were cut into sections of 4 μm and stained with Hematoxylin and Eosin (H&E). With a Phillips IntelliSite UltraFast scanner, glass slides were digitized at 20x magnification. The digital images were aligned by performing rigid and affine image alignment. The tumour and the muscularis propria (MP) were manually delineated to create 3D segmentations. In conjunction with a 3D display, the results were visualized with the Vesalius3D interactive visualization application for a 3D workstation.

**Results:**

En-bloc resection was performed in 21 BCa patients. Per case, 26–30 sections were included for the reconstruction into a 3D volume. Five cases were excluded due to export problems, size of the dataset or condition of the tissue block. Qualitative evaluation suggested an accurate registration for 13 out of 16 cases. The segmentations allowed full 3D visualization and evaluation of the spatial relationship of the BCa tumour and the MP.

**Conclusion:**

Digital scanning of en-bloc resected specimens allows a full-fledged 3D reconstruction and analysis and has a potential role to support pathologists in the staging of BCa.

## Background

Histopathological analysis is the cornerstone in bladder cancer (BCa) diagnosis. The histological grade and stage of BCa are important prognostic parameters for therapeutic decision-making [1, 2]. Patients with muscle-invasive bladder cancer have a higher risk of cancer-specific mortality compared to patients without muscle invasion [2]. Initial treatment is generally performed by transurethral resection of the bladder tumour (TURB), allowing tissue removal regardless the location or size. Histopathologic analysis of TURB specimens is limited since it is difficult to determine the degree of invasion [3]. Important landmarks for staging, such as the lamina propria or the muscularis propria (MP), are often difficult to recognize in the small TURB fragments. Moreover, these fragments are often subject to thermal artifacts or tangential sectioning [4]. Both factors result in a poor interobserver agreement of BCa staging [5]. Furthermore, BCa is often understaged at the initial resection because of the absence of MP in the resection specimen [6]. As a result, upstaging to a muscle-invasive tumour after a second TURB occurs in 10–20% [2] of BCa. To improve staging, en-bloc resections, in which the bladder tumour is resected as a whole are increasingly performed [3]. Previous studies including en-bloc resections have reported to contain MP in 96–100% of cases [3, 7, 8]. In en-bloc resections, a better impression of the spatial arrangement of the tissue can be obtained, assisting the pathologists with the orientation [3]. In general, pathologists examine an average number of 1–2 slides per tissue block, depending on the type of tissue [9]. These sections are only a small fraction of a the three-dimensional (3D) resected specimen. As only a fraction of heterogeneous tissue is examined, undersampling of the BCa specimen is likely to occur. To enable a pathologist to study a complete tissue sample comprehensively, it is necessary to cut and stain multiple consecutive tissue slides. However, it is impractical and time consuming to visualize and analyze a large number of tissue slides per sample by conventional microscopy.

Whole slide image (WSI) systems provide pathologists histopathologic glass slides as digital high resolution images with magnifications similar to conventional microscopy. WSI offers the possibility to create a digital 3D reconstruction based on stacks of two-dimensional (2D) WSIs. 3D reconstructed histology images hold the potential to assist pathologists in visualizing the 3D spatial arrangements of structures. One of the major problems encountered in 3D image reconstruction of histology slides is tissue deformation due to fixation, cutting, and mounting of the specimen during sample preparation [10–14]. Several methods have been proposed to align multiple 2D WSIs to create a 3D image [10–16]. To our knowledge, the 3D digital reconstruction of BCa tumours from histological slides has not been described before.

In this study, we present a method of preparing, reconstructing and visualizing 3D images reconstructed from multiple consecutive 2D WSIs of BCa en-bloc specimens. Moreover, we introduce a technology that enables interactive manipulation of 3D histological volumes. Additionally, we describe a method to visualize the tumour and MP, without the submucosal layer, allowing to review the spatial relationship of these structures.

## Methods

### Tissue preparation

Formalin fixed, paraffin-embedded tissue blocks from en-bloc TURB specimens were retrieved from the archive of the Amsterdam UMC, location Academic Medical Center (AMC), the Netherlands. The study was approved by Institutional Review Board of the AMC, Amsterdam (W16_088 # 16.108). Fifty-one sequential sections of 4–5 μm were cut using a microtome. Every second slide was mounted onto a glass slide, leaving the possibility to use the adjacent section for different stains or other imaging modalities.

The mounting protocol, as extension on the protocol created by Soufan et al. was aimed to minimize folding and deformation of the tissue [17]. In short, the tissue section was kept horizontal by placing the section on a drop of water on a glass slide. The glass slides were placed on a hot plate of 42 °C for 2 min, allowing the section to stretch. Using a vacuum pump, water was gently removed. The glass slide was placed on a hot plate of 32 °C for 30 min to let the remaining water evaporate. The slides were dried for at least 2 h at 37 °C and stained with Hematoxylin and Eosin (H&E). Smoothelin (Biocare medical, 1:200 dilution, clone R4A) was used on an additional slide to visualize the smooth muscle bundles of the MP [18]. The slides were stained using an automated slide preparation system (Benchmark Ultra, Ventana). The signal detection was performed with a biotin-free OptiView DAB IHC detection kit (Ventana Medical Systems).

If present, the BCa was staged according to the TNM classification system and the World Health Organization (WHO) 1997 and 2004 grading system [19].

### Scanning

The glass slides were digitized using the Phillips IntelliSite UltraFast Scanner (Philips Digital Pathology Solutions, Best, the Netherlands) and were exported as a TIFF file at 20x magnification, resulting in images with a resolution of 0.5 μm/pixel. WSIs with tissue folds or out-of-focus areas were excluded. When slides were excluded, a neighboring slide was doubled to have a good representation of the distance between slides.

### Alignment

A pair-wise reconstruction of the bladder tumour was performed by aligning the 2D WSIs, starting the alignment with the center slide. The datasets were aligned using the Elastix toolbox [20], by performing coarse-to-fine multi-resolution rigid Euler transformation followed by an affine transformation and a subsequent multi-scale b-spline transformation. To speed up the registration, the alignment was performed on 10x downsampled grayscale images. The resulting transformations were applied on the high-resolution images. The normalized correlation coefficient metric was used for the similarity measure. Deformation during the b-spline transformation was restricted by not only taking into account the similarity into the cost-function, but also limit the amount of bending by introducing a bending energy penalty term. Via this term, the sum of the second order spatial derivatives of the transformation, sharp deviations of the transformation are penalized.

### Segmentation and visualization

**Fig. 1:**
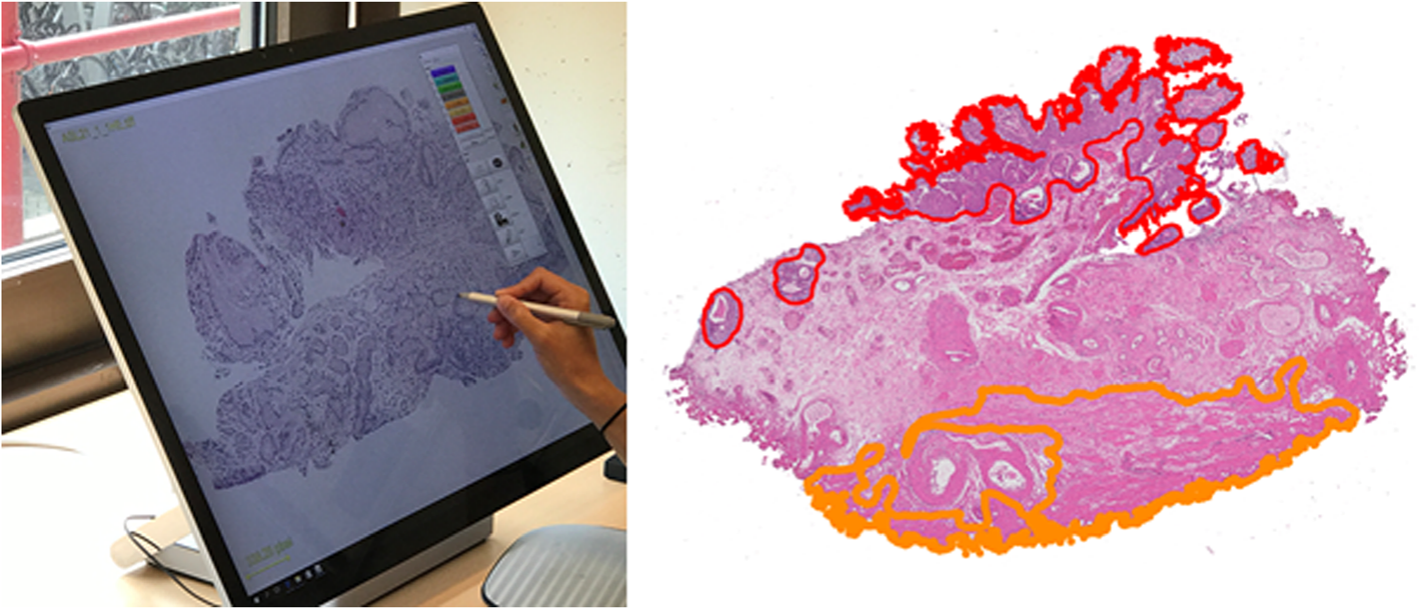
Left: Impression of the software and hardware used for delineation of the digitized tissue slides. Right: digitized en-bloc resection specimen with manual delineations. The tumour has been delineated in red, the MP in orange

The BCa and the MP were manually delineated by an experienced observer (IJ) using in-house developed software [21] (Fig. 1) operated on a computer with 28 in. touchscreen (Microsoft Surface Studio, Microsoft, Redmond, USA). These delineations were subsequently checked and corrected by a specialized urinary tract pathologist (CDSH). The segmentations of different structures allow the user to highlight the structures of interest (e.g. tumour), and to see the 3D distribution.

**Fig. 2:**
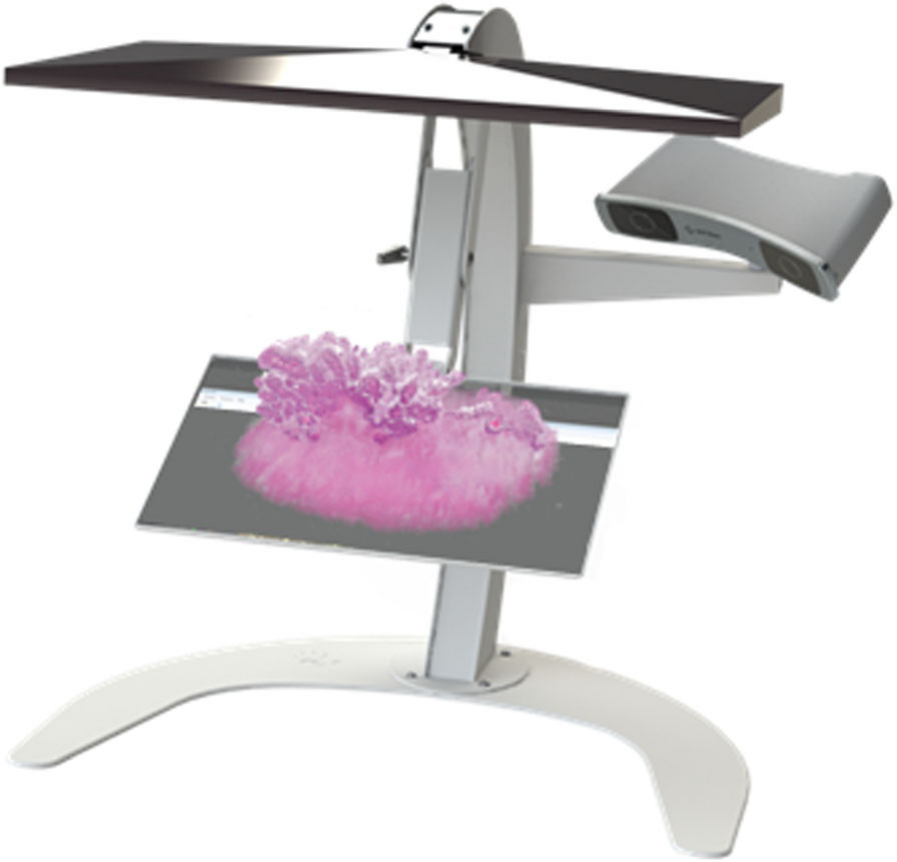
PS-Medtech C-station with a 3D reconstruction of an en-bloc bladder tumour resection, allowing manual manipulation of the data

The aligned dataset was imported into a 3D workstation (C-Station, PS-Medtech, Amsterdam, NL) (Fig. 2) and visualized using an interactive application (Vesalius3D, PS-Medtech, Amsterdam, NL). Using optical tracking, footswitches and live 3D rendering, users interact with their data as if they are holding the data in their hands. Thus 3D data volumes can be rotated, zoomed, and virtually sectioned through multiple planes of the data volume.

### Qualitative assessment of the registration performance

The accuracy of the alignment of 2D slices to create a 3D volume was qualitatively assessed by two expert observers (IJ, ML) by viewing the 3D renderings. Special attention has been devoted to tissue borders and the course of larger structures (e.g. the lumen of a blood vessel). Misalignment errors were seen as discontinuities between slides.

## Results

### Samples

**Fig. 3:**
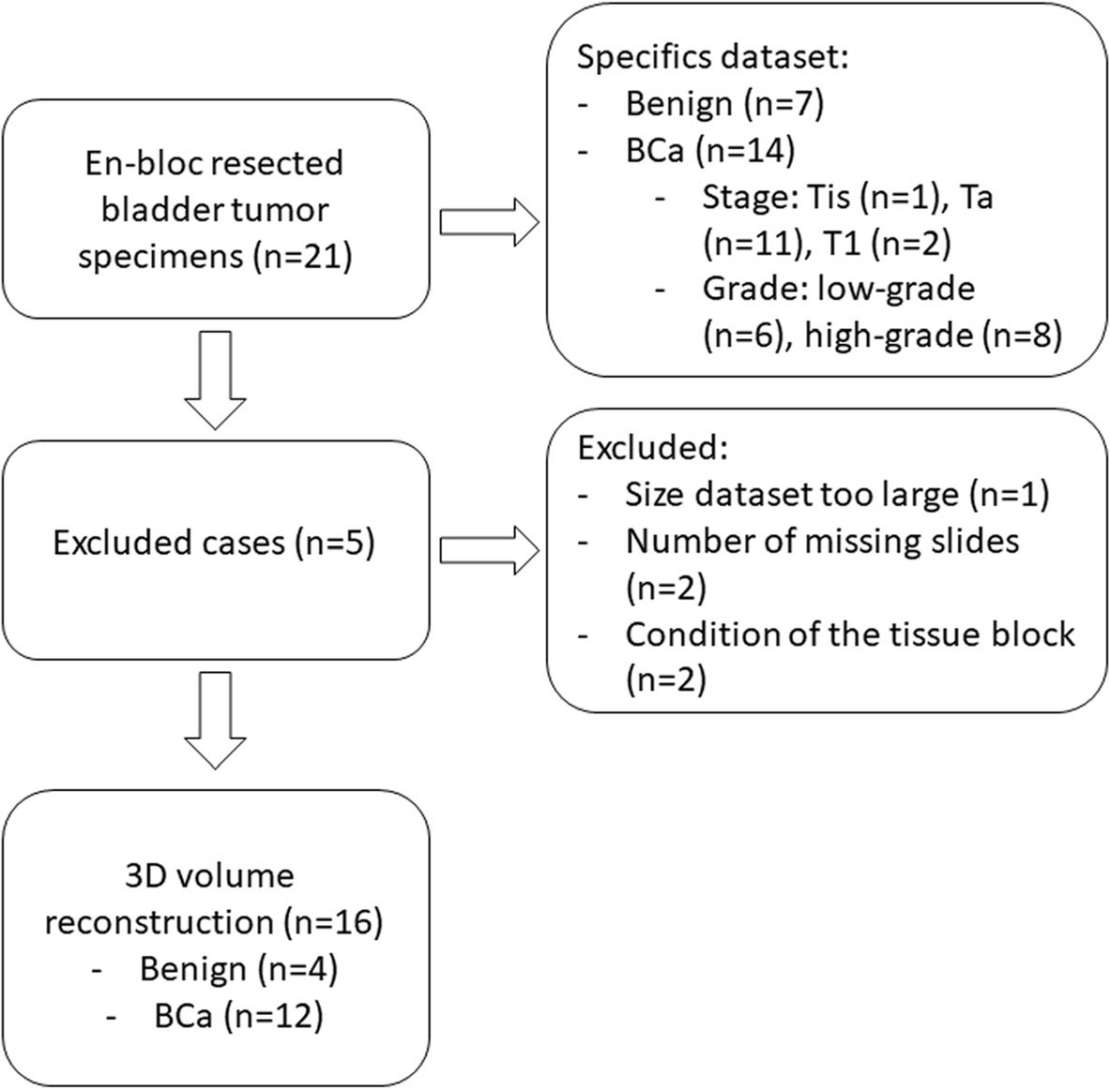
Flow diagram of the included cases

Figure 3 shows the flow diagram of the included and excluded cases. A total of sixteen en-bloc resection specimens were reconstructed into a 3D volume. The most common reason of slide exclusion was slide loss due to export problems from the Philips server. The size of the datasets varied from 1.5 GB to 66 GB. The number of slides per case varied from 26 to 30. In two patients, slides were doubled due to tissue folds or out-of-focus areas. No difference between the initial staging and staging of the whole 3D reconstructions was observed.

**Fig. 4:**
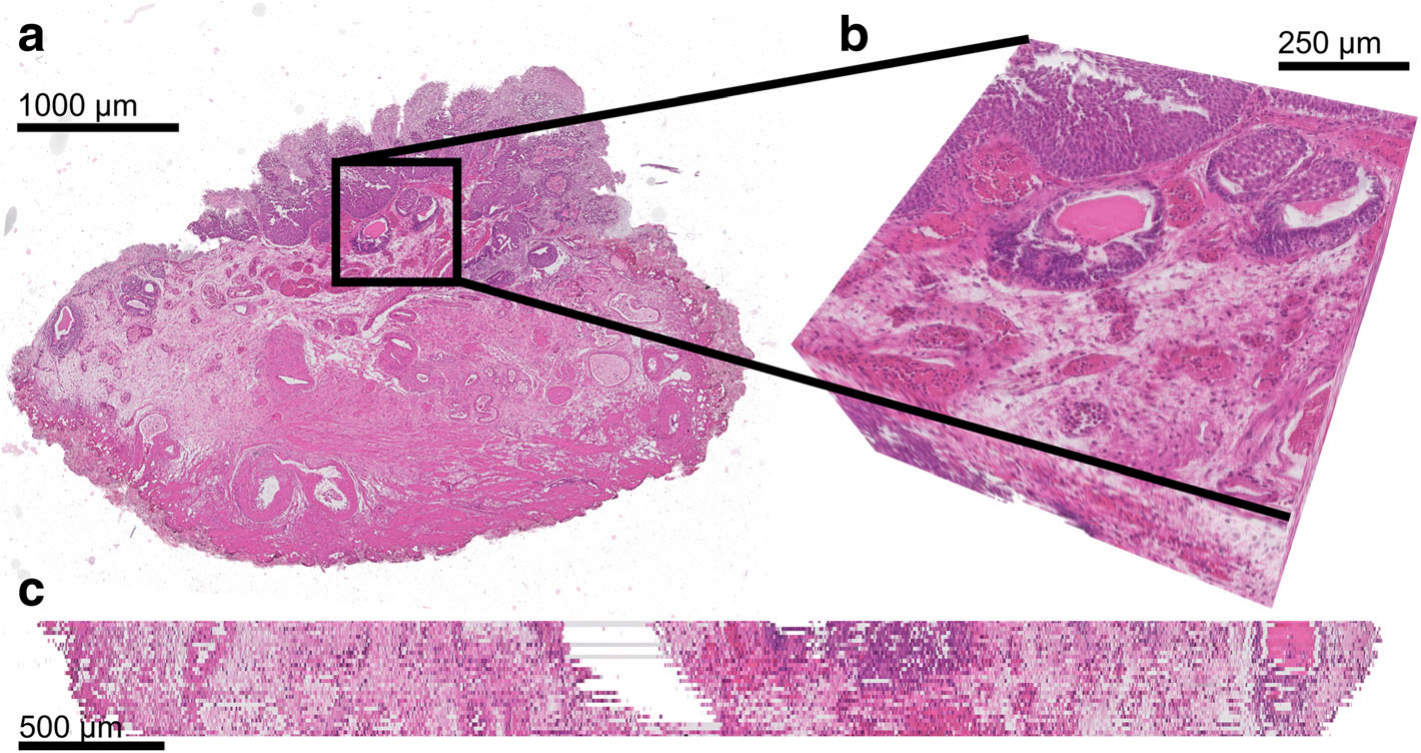
3D reconstruction of en-bloc resection specimen of patient 1 **a**. 3D rendering **b**. Detailed view showing a zoomed-in view of a lumen and tumour nests. **c**. Sagittal view, showing the alignment

**Fig. 5:**
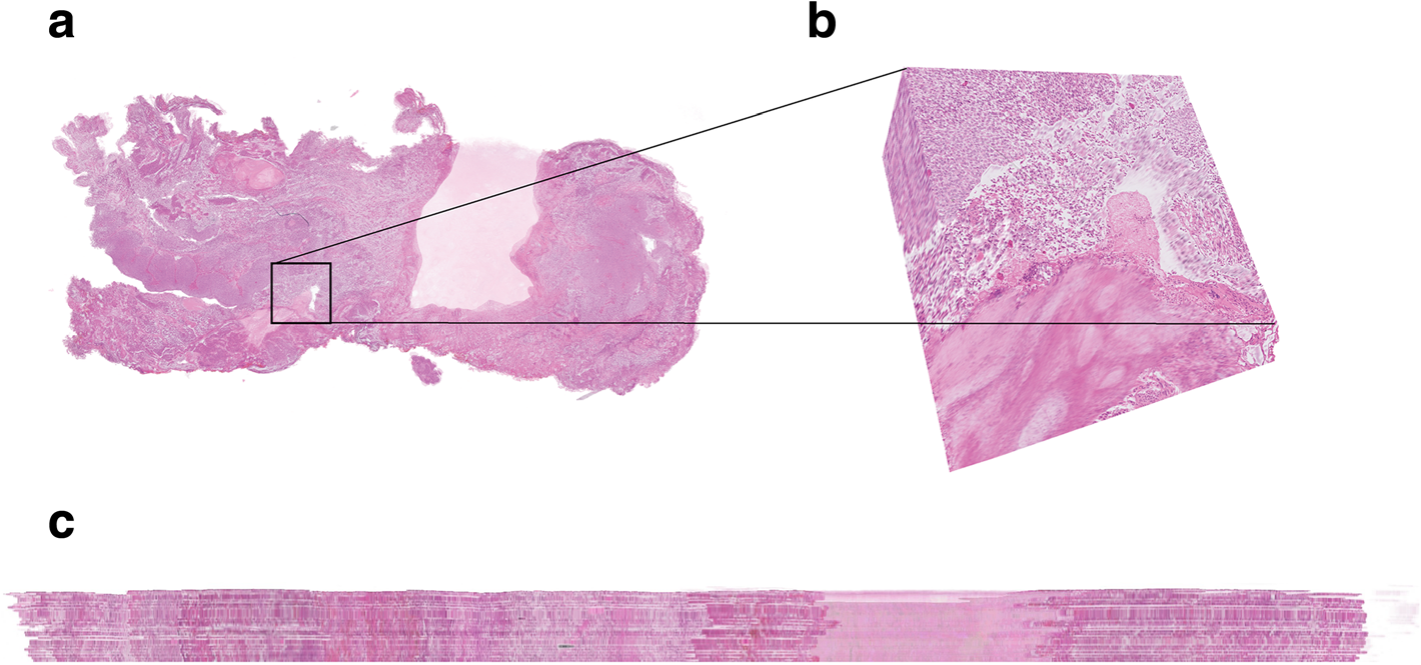
3D reconstruction of en-bloc resection of patient 21. The lamina propria is faded out in order to see the differences in tumour growth into the lamina propria. **a**. 3D rendering **b**. Detailed view showing a tumour border **c**. Sagittal view, showing the alignment

Qualitative assessment showed good alignment in 13 out of 16 cases. 3D reconstruction results of the full rendering, sagittal and detailed view of two datasets are shown in Figs. 4 and 5. Figure 4a-c demonstrates tissue of a papillary low grade (grade 2) Ta BCa with presence of the MP. The zoomed-in images are showing the high quality of the registration. Figure 5a-c shows tissue of a high grade (grade 3) T1 BCa without presence of the MP.

### Segmentation and visualization

After importing the aligned datasets into the visualization application Vesalius3D, the datasets were visualized on a 3D monitor. Through the 3D interaction on the C-Station the data could be rotated and virtually scrolled through. This way the multiple slides could be easily evaluated.

**Fig. 6:**
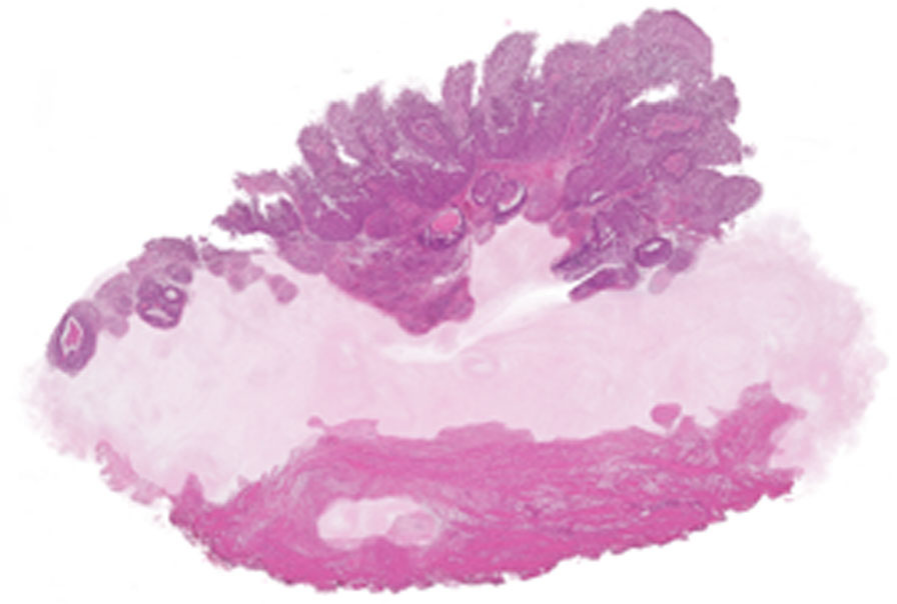
Segmentation of en-bloc resection specimen 1. The upper part shows the bladder cancer, the lower part shows the muscularis propria. The tissue between these two structures is blurred, making it possible to easily view the relationship of the two layers

Figure 6 shows a tumour in relation to the MP using the segmentations. By using the segmentations, the distance of the BCa and the MP is clearly visible in the Ta tumour. The blurred area might give the pathologist the possibility to clearly view the areas that deviate, and where a more invasive aspect is seen.

## Discussion

We have demonstrated the work-up of high quality 3D reconstructions of en-bloc TURB specimen from sequential 2D WSIs. With the provided segmentations of BCa tumour and MP, the spatial relationship between these structures could be visualized in 3D. By providing better insight in the growth pattern of a tumour, this method has the potential to assist the pathologist in accurate staging of BCa.

To our knowledge, this is the first study focusing on 3D reconstructing BCa tumours out of multiple 2D histological slides. By evaluating a 3D volume instead of the conventional 2D images, we assume that more information can be visualized. We created segmentations of the tumour and the MP and faded out the area in between, to emphasize the distance between these two layers and clarify the changes that occur throughout the different slides. Booth et al. used 3D segmentations to visualize the mammarian lobule in relation to ductal carcinoma in situ and noticed a complexity that was of higher order than was apparent from the 2D slides [13]. A small number of previous studies have created 3D reconstructions out of histological slides, using multiple sections [10–15]. Booth et al. discussed the depth of the stack for the 3D reconstruction of breast cancer and found 30 slides, with a thickness of 4 μm, to be an insufficient number for a spatial reconstruction [13]. In future, we aim to increase our reconstructions to determine the optimal number of sections to create a 3D volume. Moreover, we would like to investigate if it is necessary to use consecutive slides. Due to the anisotropic voxel (volumetric pixel) size of the 3D image, important information could be missed.

We have shown good qualitative results in the registration of multiple WSIs. In previous studies that focused on the 3D reconstruction of histological slides, various registration methods have been used [10–15]. In our study, we have only used existing open-source tools, being easily accessible. Registration of the WSIs is a challenging task since the volume of the slides within a specimen can differ enormously due to deformation of the tissue. Deformation occurs as a result of the fixation with formalin, cutting and mounting of the specimen. In our protocol, we tried to minimize deformation by adjusting the protocol and keeping the slides as horizontal as possible. Some groups used artificial fiducial markers as a reference point for the registration [14, 22]. These fiducial markers are placed alongside the specimen during embedding. However, during cutting and mounting of the specimen, non-linear deformation occurs throughout the whole specimen, and therefore external fiducial markers cannot predict this deformation for the whole tissue [15, 22]. Other groups have used structures that are visible through several planes as a reference point [10, 13]. However, especially in tumourous tissue, it is difficult to use this method for the basis of alignment because of the heterogeneity of the tumour. Another obstacle for the visualization is the staining inconsistency, which can even be noted within sections, several groups have tried to make up for the staining differences by colour normalization [23].

In our study, we only mentioned a qualitative measure for the accuracy of registration. Quantitative measures for registration are difficult to determine. Some groups have used Hausdorff evaluation based on manual delineations and concluded only minor registration errors [12, 14]. However, in our study we did not use Hausdorff evaluation due to the lack of anatomic markers in the tissue, in specific within the tumour. Thereby are these anatomic markers mostly based on manual segmentation of the marker, thereby introducing a dependency.

During the sample workup the major reason of slide loss were exportation problems from the Philips server due to the size of the images. Therefore, local storage solutions as well as network speed and infrastructures must be adapted. The FDA recently gave approval for the use of digital slides scanners in diagnostics [24]. We expect that this recent development may give a boost to the use and therefore the evolution of digital slide scanners and subsequently data storage more reliable.

A drawback of the presented methodology is the time consuming process of tissue preparation, as also reported in other studies [12, 13, 25, 26]. Sectioning and mounting of the tissue requires human labour. Onozato et al. have showed a method for automated sectioning with positive results [16, 26]. As for now, this is not common practice in pathology laboratories, since these machines still take more time than manual sectioning [26]. Furthermore, the manually delineation of the tissue structures was a time consuming process. Eventually, automatic tissue segmentation can be implemented [10]. Furthermore, we have used only a small portion of the tumors to create a 3D reconstruction since were not allowed to section entire tumor blocks. In future work we would like to use tumor specimen that we can section completely.

3D pathology has the potential to improve insight in the growth pattern of BCa and thus refine the staging. Although pathologist are trained in visualizing the spatial arrangement of a tumour based on the 2D representation of a single slide, 3D reconstructions could support when orientation of specimen is difficult to determine. Moreover, 3D reconstructions could give better insight in the suggested sub-staging of non-muscle invasive T1 BCa. In T1 BCa the 5-year progression rates vary between 21 and 50% [1]. The two most studied systems, stratification of the muscularis mucosa invasion (T1a versus T1b) and assessing the amount of invasion into the lamina propria (T1e and m), show an increased prognostic value compared to normal T1 staging [27]. The main objection of not implementing the T1a/b sub-staging system in clinical practice is the discontinuous nature of the muscularis mucosae and interobserver variability [28]. The T1e/m staging system however, proved to be a more consistent method [29, 30]. We believe by implementing the 3D reconstructions of en-bloc bladder resection specimen into clinical practice, the visualization of the possible tumour ingrowth can be simplified. In the future, we will focus on the clinical relevance of creating 3D reconstructions for routine diagnosis. Eventually, we need to assess the added value of 3D reconstructions, together with optimizing visualization techniques. Moreover, this could be of use in other fields, such as follicular thyroid neoplasms, to determine invasion depth.

## Conclusion

In conclusion, we have created a 3D reconstruction of a BCa tumour out of multiple 2D WSIs. 3D reconstructions show added value of tumour configurations and may improve insight into the architectural changes and refine the classification of BCa by providing detailed spatial and structural information to the assessment by light microscopy.

## Abbreviations

2D: Two-dimensional
3D: Three-dimensional
BCa: Bladder cancer
CIS: Carcinoma in situ
H&E: Hematoxylin & Eosin
MP: Muscularis propria
TURB: Transurethral resection of bladder tumour
WSI: Whole slide imaging

## References

[1] Babjuk M, Böhle A, Burger M, Capoun O, Cohen D, Compérat EM, et al. EAU guidelines on non–muscle-invasive urothelial carcinoma of the bladder: update 2016. Eur Urol 2016;1–15. Available from: http://linkinghub.elsevier.com/retrieve/pii/S0302283816302494 10.1016/j.eururo.2016.05.041 27324428

[2] Witjes JA, Compérat E, Cowan NC, De Santis M, Gakis G, James N, et al. Muscle-invasive and metastatic bladder cancer. Eur Urol Guidel. 2015. ISBN:9789079754656 https://uroweb.org/guideline/bladder-cancer-muscle-invasive-and-metastatic/.

[3] Muto G, Collura D, Giacobbe A, D’Urso L, Muto GL, Demarchi A, et al. Thulium:yttrium-aluminum-garnet laser for en bloc resection of bladder cancer: clinical and histopathologic advantages. Urology; 2014;83(4):851–855. Elsevier Inc. Available from: 10.1016/j.urology.2013.12.022 10.1016/j.urology.2013.12.022 24548711

[4] Cheng L, Montironi R, Davidson DD, Lopez-Beltran A (2009). Staging and reporting of urothelial carcinoma of the urinary bladder. Mod Pathol Nat Publ Group.

[5] Compérat E, Egevad L, Lopez-Beltran A, Camparo P, Algaba F, Amin M, et al. An interobserver reproducibility study on invasiveness of bladder cancer using virtual microscopy and heatmaps. Histopathology. 2013;63:756–66 Available from: https://onlinelibrary.wiley.com/doi/full/10.1111/his.12214. 10.1111/his.12214 24102813

[6] Gendy R, Delprado W, Brenner P, Brooks A, Coombes G, Cozzi P (2016). Repeat transurethral resection for non-muscle-invasive bladder cancer: a contemporary series. BJU Int.

[7] Naselli A, Puppo P (2017). En bloc transurethral resection of bladder tumors: a new standard?. J Endourol.

[8] Kramer MW, Rassweiler JJ, Klein J, Martov A, Baykov N, Lusuardi L (2015). En bloc resection of urothelium carcinoma of the bladder (EBRUC): a European multicenter study to compare safety, efficacy, and outcome of laser and electrical en bloc transurethral resection of bladder tumor. World J Urol.

[9] Buesa RJ. Productivity standards for histology laboratories. Ann Diagn Pathol; 2010;14(2):107–124. Elsevier Inc.. Available from: 10.1016/j.anndiagpath.2009.12.005 10.1016/j.anndiagpath.2009.12.005 20227016

[10] Norton KA, Namazi S, Barnard N, Fujibayashi M, Bhanot G, Ganesan S (2012). Automated reconstruction algorithm for identification of 3D architectures of cribriform ductal carcinoma in situ. PLoS One.

[11] Magee D, Song Y, Gilbert S, Roberts N, Wijayathunga N, Wilcox R, et al. Histopathology in 3D: from three-dimensional reconstruction to multi-stain and multi-modal analysis. J Pathol Inf. 2015;6 Available from: http://www.ncbi.nlm.nih.gov/pmc/articles/PMC4355830/. 10.4103/2153-3539.151890 PMC4355830 25774317

[12] Roberts N, Magee D, Song Y, Brabazon K, Shires M, Crellin D (2012). Toward Routine Use of 3D Histopathology as a Research Tool. Am J Pathol.

[13] Booth ME, Treanor D, Roberts N, Magee DR, Speirs V, Hanby AM (2015). Three-dimensional reconstruction of ductal carcinoma in situ with virtual slides. Histopathology..

[14] Song Y, Treanor D, Bulpitt AJ, Magee DR. 3D reconstruction of multiple stained histology images. J Pathol Inf. 2013;4 Available from: http://www.pubmedcentral.nih.gov/articlerender.fcgi?artid=3678754&tool=pmcentrez&rendertype=abstract. 10.4103/2153-3539.109864 PMC3678754 23766943

[15] Marchiò C, Sapino A, Arisio R, Bussolati G (2006). A new vision of tubular and tubulo-lobular carcinomas of the breast, as revealed by 3-D modelling. Histopathology..

[16] Onozato ML, Klepeis VE, Yagi Y, Mino-Kenudson M (2012). A role of three-dimensional (3D)-reconstruction in the classification of lung adenocarcinoma. Anal Cell Pathol.

[17] Soufan AT, Ruijter JM, van den Hoff MJB, de Boer PAJ, Hagoort J, Moorman AFM (2003). Three-dimensional reconstruction of gene expression patterns during cardiac development. Physiol Genomics.

[18] Maake C, Landman M, Wang X, Schmid DM, Ziegler U, John H (2006). Expression of smoothelin in the normal and the overactive human bladder. J Urol.

[19] Moch H, Humphrey P, Ulbright T, Reuter V.E. (Eds): WHO Classification of Tumours of the Urinary System and Male Genital Organs (4th edition). IARC: Lyon 2016. ISBN: 978-92-832-2437-2.

[20] Klein S, Staring M, Murphy K, Viergever MA, Pluim J (2010). Elastix: a toolbox for intensity-based medical image registration. IEEE Trans Med Imaging.

[21] Kamphuis G, de Bruien D, Brandt M, Knoll T, Conort P, Lapini A (2016). Comparing image perception of bladder Tumours in four different Storz professional image enhancement system ( SPIES ) modalities using the íSPIES app. J Endourol.

[22] Boag AH, Kennedy LA, Miller MJ (2001). Three-dimensional microscopic image reconstruction of prostatic adenocarcinoma. Arch Pathol Lab Med..

[23] Clarke EL, Treanor D. Colour in digital pathology: a review. Histopathology. 2016:153–63. 10.1111/his.13079 27607349

[24] Evans AJ, Salama ME, Henricks WH, Pantanowitz L. Implementation of whole slide imaging for clinical purposes issues to consider from the perspective of early adopters. Arch Pathol Lab Med. 2017;141(7):944–59. 10.5858/arpa.2016-0074-OA 28440660

[25] Clarke GM, Eidt S, Sun L, Mawdsley G, Zubovits JT, Yaffe MJ (2007). Whole-specimen histopathology: a method to produce whole-mount breast serial sections for 3-D digital histopathology imaging. Histopathology..

[26] Onozato ML, Hammond S, Merren M, Yagi Y (2013). Evaluation of a completely automated tissue-sectioning machine for paraffin blocks. J Clin Pathol.

[27] van de Fransen Putte EE, Behrendt MA, Pigot GLS, van der Kwast TH, van Rhijn BWG. Prognostic significance of substage and WHO classification systems in T1 urothelial carcinoma of the bladder. Curr Opin Urol. 2015;1 Available from: https://insights.ovid.com/pubmed?pmid=26125511. 10.1097/MOU.0000000000000202 26125511

[28] Ro JY, Ayala AG, El-Naggar A (1987). Muscularis mucosa of urinary bladder. Am J Surg Pathol.

[29] Van Rhijn BWG, Van Der Kwast TH, Alkhateeb SS, Fleshner NE, Van Leenders GJLH, Bostrom PJ (2012). A new and highly prognostic system to discern T1 bladder cancer substage. Eur Urol.

[30] Bertz S, Denzinger S, Otto W, Wieland WF, Stoehr R, Hofstaedter F (2011). Substaging by estimating the size of invasive tumour can improve risk stratification in pT1 urothelial bladder cancer-evaluation of a large hospital-based single-Centre series. Histopathology..

